# Validity and Reproducibility of the Iodine Dietary Intake Questionnaire Assessment Conducted for Young Polish Women

**DOI:** 10.3390/ijerph14070700

**Published:** 2017-06-29

**Authors:** Dominika Głąbska, Ewa Malowaniec, Dominika Guzek

**Affiliations:** 1 Department of Dietetics, Faculty of Human Nutrition and Consumer Sciences, Warsaw University of Life Sciences (WULS-SGGW), 02-787 Warsaw, Poland; ewa_malowaniec@sggw.pl; 2 Department of Organization and Consumption Economics, Faculty of Human Nutrition and Consumer Sciences, Warsaw University of Life Sciences (WULS-SGGW), 02-787 Warsaw, Poland; dominika_guzek@sggw.pl

**Keywords:** iodine, food frequency questionnaire, validation study, validity, reproducibility, young women

## Abstract

The aim of this study was to analyse a designed brief iodine dietary intake questionnaire based on a food frequency assessment (IOdine Dietary INtake Evaluation-Food Frequency Questionnaire—IODINE-FFQ), including the assessment of validity and reproducibility in a group of 90 Polish women aged 20–35 years. Participants collected 3-day dietary records and filled in the IODINE-FFQ twice (FFQ1—directly after the dietary record and FFQ2—6 weeks later). The analysis included an assessment of validity (comparison with the results of the 3-day dietary record) and of reproducibility (comparison of the results obtained twice—FFQ1 and FFQ2). In the analysis of validity, a Bland-Altman index of 5.5% and 4.4% was recorded, respectively for FFQ1 and FFQ2. In the analysis of reproducibility it was 6.7%, but the share of individuals correctly classified into tertiles was over 70% (weighted κ of 0.675). It was stated, that assessment of IODINE-FFQ revealed a satisfactory level of validity and reproducibility in the analysis of Bland-Alman plot. The IODINE-FFQ may be indicated as a tool for the assessment of iodine intake in the young women in Poland, however further studies should be considered in order to obtain the practical tool for public health specialists. Due to the lack of validated iodine-specific food frequency questionnaires for countries of Eastern Europe, the IODINE-FFQ may be adjusted for courtiers other than Poland including iodine-fortified products.

## 1. Introduction

The analysis conducted by the World Health Organization (WHO) [1] in 2003 indicated, that worldwide frequency of inadequate iodine intake was high—35.2% of general population was characterized by insufficient iodine intake. Moreover, only Americas and Western Pacific were regions of lower frequency of inadequate iodine intake, whereas in Europe, the frequency was the highest—52.7% of inhabitants, differing from 42.6% (Western Europe) to 59.9% (Eastern Europe) [1]. In the succeeding studies the frequency of iodine insufficient intake was a little bit lower, but still alarming, as in 2007 it was 30.6% for the world and 52.0% for Europe [2] and in 2011 it was 28.5% for the world and 44.2% for Europe [3]. Currently, for Poland, mild population iodine deficiency is indicated [4], like for 14 other European countries, including Italy [5], France [6], Belgium [7] and Denmark [8].

The adverse effects of iodine deficiency, being associated with inadequate thyroid hormone production, may affect individuals at all age, but women of childbearing age, pregnant women and children are a special target groups indicated to increase iodine intake, in order to reduce fetal and perinatal mortality, as well as to improve motor and cognitive performance of the offspring [9]. 

The strategy for the iodine deficiency disorders, recommended by WHO [1], includes supplementation and food fortification, as well as populations surveillance involving monitoring, evaluation, correction of iodine deficiency and education. As the need of educating young women about the most important dietary iodine sources is emphasized [10], all the tools enabling it are of a great value. To achieve the target of increasing iodine intake by educating about the sources of mentioned nutrient, the practical tool to assess the iodine intake quickly and with the highest possible accuracy may be a solution, while ensuring the ease of use and ease of interpretation of the data.

For correction of iodine intake and dietary iodine intake monitoring, the information about accurate absolute intake is not essential and estimated iodine intake information is sufficient, so the food frequency questionnaires could be the good method enabling quick assessment [11]. Also the European Community’s ‘EURopean micronutrient RECommendations Aligned (EURRECA)’ Network of Excellence indicated, that food frequency questionnaires may be perceived as a valid method for assessing mineral intake, particularly for calcium, but also for iodine and zinc [12]. 

In the review of Serra-Majem et al. [12], the number of nine validation studies of iodine intake assessment methods published until March 2008 was indicated. In the aspect of iodine deficiency, especially important are iodine-specific food frequency questionnaires, as questionnaire validated in Denmark [13] or Great Britain [14]. During the following years, other iodine-specific food frequency questionnaires were also validated for Great Britain [15], Australia [16], or Malaysia [17]. Such a validated questionnaires, as previously mentioned, not only enable assessment of intake, but also have considerable advantages, such as accuracy, confirmed validity, and economical aspects of conducting studies [12]. As a result, it may be indicated, that not only single assessment of iodine intake is possible while iodine-specific food frequency questionnaires are applied, but also repeated assessments in the period of time in a larger populations, to obtain the aims of health promoting policy.

However, for the countries of Eastern Europe (according to the WHO *inter alia*: Belarus, Bulgaria, Czech Republic, Hungary, Poland, Republic of Moldova, Romania, Russian Federation, Slovakia, Ukraine), being characterized by the highest frequency of inadequate iodine intake [1], no such validation was conducted. Moreover, the validation of Polish multiple-nutrients food frequency questionnaire, including 31 nutrients, conducted by Kowalkowska et al. [18] in the group of Polish young women, did not include iodine intake assessment. Taking it into account, elaborating and validating the iodine-specific food frequency questionnaire for Eastern Europe populations would be a promising direction in the practical implementing of the health promoting strategies.

The aim of the presented study was to assess the validity and reproducibility of the designed iodine dietary intake questionnaire based on a food frequency assessment (IOdine Dietary INtake Evaluation-Food Frequency Questionnaire–IODINE-FFQ) in a group of Polish women aged 20–35 years.

## 2. Materials and Methods

The study was conducted according to the guidelines laid down in the Declaration of Helsinki, and all procedures involving human subjects were approved by the Bioethical Commission of the National Food and Nutrition Institute in Warsaw (No. 0701/2015).

### 2.1. Designing an Iodine Dietary Intake Questionnaire: Iodine Dietary Intake Evaluation-Food Frequency Questionnaire (IODINE-FFQ)

The designed IODINE-FFQ was based on a food frequency assessment. Only food products that were sources of iodine were taken into account. All food products characterised by iodine content no lower than 0.1 µg per 100 g were chosen on the basis of Polish food composition tables, elaborated by the National Food and Nutrition Institute in Warsaw [19], being a member of the EuroFIR, taking part *inter alia* in work package associated with nutritional value of traditional food products [20]. 

All food products meeting the assumed criteria were grouped into 13 food product groups and 44 related sub-groups characterised by a similar range of iodine content, as presented in Table 1. Due to the fact that in a typical Polish diet, the main iodine sources are dairy products and fish products [21], 10 dairy product sub-groups and eight fish product sub-groups were included in the questionnaire. 

The iodine-fortified salt, being the only iodine-fortified food product in Poland, obligatory since 1997 [22] was also included. The clustering procedure was conducted, similarly as in the previous studies [23,24], in order to obtain a lower number of items included in the questionnaire. The most popular serving sizes were determined on the basis of the Polish food model booklet [25] and verified by dietitian during the pilot research. For each group of food products, the average iodine content in a serving was specified, as is presented in Table 2 [19]. Information about the iodine quantity in the serving was not placed in the IODINE-FFQ (Table 1) in order to not interfere in providing the answers, but the serving sizes were specified in the IODINE-FFQ both in grams and serving sizes. 

Individuals were asked about the exact number of servings of products from the groups specified in the IODINE-FFQ which had been consumed during a typical week throughout the previous year (open-ended question), similarly, as in the case of previous own studies conducted for vitamin D [23] and iron [24]. Such approach is associated with the fact, that the dietary intake may differ throughout the year that results from the seasonality [12]. 

Participants were asked to indicate the servings of products consumed and added to consumed dishes. In the questionnaire the participants declared the typical number of servings of each product (being able to indicate not only whole integers but also decimal parts). 

During the analysis, the number of servings was divided into 7 days a week to obtain the daily number of servings. The iodine intake from the products was estimated using the following formula: iodine intake (µg) = daily number of servings × typical iodine content in one serving (Table 2). The total daily iodine intake was obtained as the sum of the values of iodine intake from all groups of products.

### 2.2. Validation of the IODINE-FFQ

**Figure 1:**
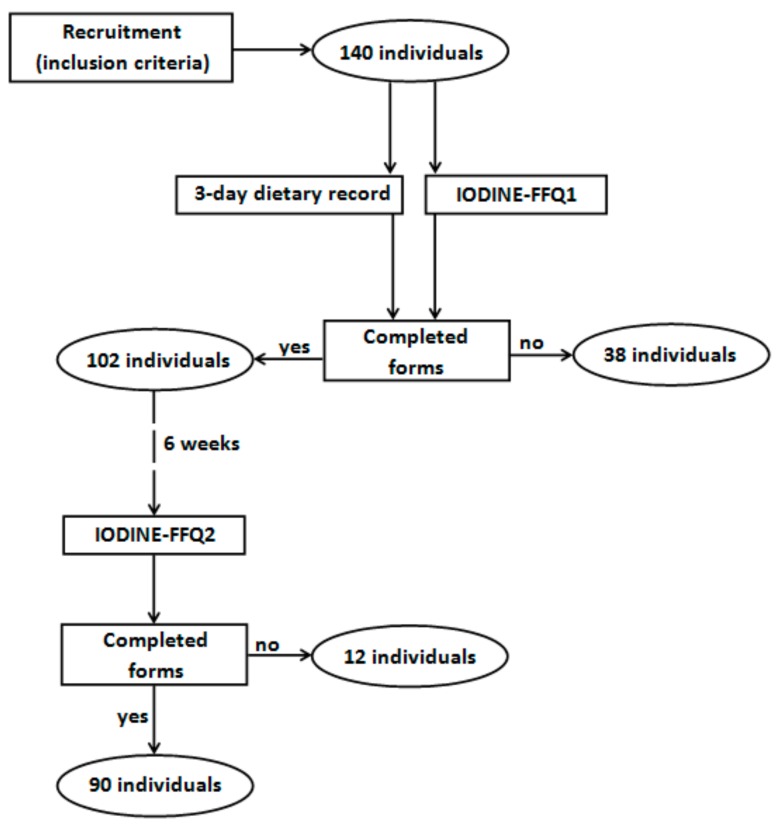
Recruitment of participants to the study.

The IODINE-FFQ was validated in a group of young women. The invitation to participate in the validation of the IODINE-FFQ as well as information about the inclusion criteria were distributed via social media. The inclusion criteria were as follows: women, aged 20–35 years, not undergoing body mass reduction or on any special diet, not pregnant and not during lactation, without any chronic diseases, and living in Warsaw. There were 140 individuals meeting the inclusion criteria who volunteered to participate in the study. Finally, validation of the assessed IODINE-FFQ was conducted in a group of 90 young women, as 50 of the individuals who had initially volunteered did not complete all of the required elements (Figure 1). The obtained sample size was in accordance with recommendations on sample size for validation studies of food frequency questionnaires, as the guidelines state that at least 50 to 100 subjects for each demographic group is recommended [11]. 

The study of validation was conducted in autumn, during a period of 3 months—from September to November. During this period the participants were asked to conduct 3-day dietary records and to fill in the IODINE-FFQ twice (FFQ1—filled in directly after conducting the 3-day dietary record and FFQ2—filled in 6 weeks after the FFQ1). 

Validation of the obtained IODINE-FFQ questionnaire was conducted according to the same methodology, as validations published previously [23,24]. It included an analysis of the validity (external validation comparing results of the FFQ1 and FFQ2 with results of the 3-day dietary record, whereas both assessments were conducted by the same researcher) and reproducibility of the method (internal validation comparing results obtained twice—FFQ1 and FFQ2, with both assessments conducted by the same researcher), as defined by Willett and Lenart [26]. Both the 3-day dietary record and the IODINE-FFQ assessments were based on self-reported data. Due to the fact, that the aim of the study was to validate the questionnaire that enables assessment of dietary iodine intake, the reference value was to be not the iodine status, but the iodine intake. Similar approach was taken in the case of a number of validation studies, as dietary record was indicated as a “gold standard” for such assessments in the review of 109 studies assessing intake of various minerals [12]. In the case of iodine especially important is to disassociate the issue of dietary iodine intake and the total iodine intake, as apart from the dietary intake, other source may be the intake from air [27] that is becoming more relevant in the case of the marine areas [28]. 

For the 3-day dietary record, the basis of the analysis was the record conducted in three typical random and not successive days (2 weekdays and 1 day of the weekend). The dietary record was conducted on the basis of widely accepted and applied rules—using a structured format, with additional questions about name of the meal, time and location of consumption, meal ingredients and weight of serving (while weighted using kitchen scale) or size of serving (while estimated using standard household measures) [23,29]. To provide reliable estimates of food intake, participants were instructed on the principles of making the dietary record as well as on the necessity of accurate and scrupulous recording of all food products consumed and beverages drunk, while the serving sizes were verified afterwards by a dietitian using the Polish food model booklet [25]. The iodine intake was analysed using the Polish dietician software—“Dietetyk 2” (National Food and Nutrition Institute, Warsaw, Poland, 2001) and the Polish database of the nutritional value of products [19].

### 2.3. Statistical Analysis

The statistical analysis of validation included:

- Analysis of the Bland-Altman plots in the assessment of validity (IODINE-FFQ1 vs. 3-day record; IODINE-FFQ2 vs. 3-day record) and of reproducibility (IODINE-FFQ1 vs. IODINE-FFQ2)—the results were interpreted using the Bland-Altman index, whereas the limit of agreement values (LOA) were calculated as the sum of the mean absolute differences of iodine intake measured by the two methods, and the ± standard deviation of the absolute difference of iodine intake recorded for the two methods magnified by 1.96. In the analysis conducted with the Bland-Altman method to assess agreement between the measurements, a Bland-Altman index of a maximum of 5% (95% of individuals observed to be beyond the LOA) was interpreted, as commonly assumed [30], as positive validation of the method of measurement.

And five additional elements:

- Calculation of the root mean square errors of prediction (RMSEP) and median absolute percentage errors (MdAPE) of iodine intake in the assessment of validity (IODINE-FFQ1 vs. 3-day record; IODINE-FFQ2 vs. 3-day record) and of reproducibility (IODINE-FFQ1 vs. IODINE-FFQ2).
- Assessment of the share of individuals classified into the same tertile and misclassified (classified into opposite tertiles) in the assessment of validity (IODINE-FFQ1 vs. 3-day record; IODINE-FFQ2 vs. 3-day record) and of reproducibility (IODINE-FFQ1 vs. IODINE-FFQ2).
- Calculation of the weighted κ statistic with linear weighting to indicate the level of agreement between the classifications into tertiles in the assessment of validity (IODINE-FFQ1 vs. 3-day record; IODINE-FFQ2 vs. 3-day record) and of reproducibility (IODINE-FFQ1 vs. IODINE-FFQ2)—according to the criteria of Landis and Koch [31], values <0.20 were treated as slight agreement, 0.21–0.40—as fair, 0.41–0.60—as moderate, 0.61–0.80—as substantial, and 0.81–1.0—as almost perfect agreement.
- Assessment of the share of individuals classified into the same category (both of either adequate or inadequate intake) and of the conflicting intake adequacy category (adequate intake and inadequate intake) in the assessment of validity (IODINE-FFQ1 vs. 3-day record; IODINE-FFQ2 vs. 3-day record) and of reproducibility (IODINE-FFQ1 vs. IODINE-FFQ2). The adequate intake was defined according to the Polish recommendations for women on the Estimated Average Requirement (EAR) level as 95 µg [32].
- Analysis of the correlations between results obtained in the assessment of validity (IODINE-FFQ1 vs. 3-day record; IODINE-FFQ2 vs. 3-day record) and of reproducibility (IODINE-FFQ1 vs. IODINE-FFQ2)—the normality of distribution of the results was analysed using the Shapiro-Wilk test and then Spearman’s rank correlation was applied for nonparametric distribution.

The level of significance was accepted as *p* ≤ 0.05. Statistical analysis was carried out using Statistica software version 8.0 (StatSoft Inc., Tulusa, OK, USA) and Bland-Altman Statistica software macro by Matt Coates, version 2009 (StatSoft Inc., Tulusa, OK, USA).

## 3. Results

Iodine intake calculated in the analysed group, using the 3-day dietary record and IODINE-FFQ conducted twice (IODINE-FFQ1, IODINE-FFQ2), is presented in Table 3. While using 3-day dietary record, the observed iodine intake for the majority of the analysed group was stated to be inadequate (lower than the Estimated Average Requirement level of 95 µg a day), but while using IODINE-FFQ, it was stated to be inadequate for half of the analysed group.

The share of individuals classified into the same tertile in the validation of the IODINE-FFQ as well as the weighted κ statistic are presented in Table 4. The highest share of individuals classified into the same category (85.56%), accompanied by the lowest share of misclassified individuals (14.44%), was stated for the IODINE-FFQ1 vs. IODINE-FFQ2 comparison (assessment of reproducibility). Simultaneously, in the assessment of reproducibility the weighted κ statistic indicated substantial agreement (0.675), while in the assessment of validity it indicated slight agreement. 

The calculated RMSEP of iodine intake estimation in the assessment of validity in comparison with the results of the 3-day dietary record for the analysed IODINE-FFQ was 110.53 µg (for IODINE-FFQ1) and 99.94 µg (for IODINE-FFQ2). Simultaneously, the MdAPE of iodine intake for a comparison with the 3-day dietary record was 110.44% (for IODINE-FFQ1) and 119.14% (for IODINE-FFQ2). In the assessment of reproducibility, the RMSEP of iodine estimation, while the food frequency assessment was conducted twice during a period of 6 weeks, was 35.34 µg. The MdAPE of iodine intake for the comparison between IODINE-FFQ1 and IODINE-FFQ2 was 20.89%.

The analysis of correlation, in the assessment of validity, in comparison with 3-day dietary record, revealed significant correlation both for IODINE-FFQ1 (*p* = 0.0079; R = 0.2618) and IODINE-FFQ2 (*p* = 0.0491; R = 0.2081). Also in the assessment of reproducibility, the correlation between IODINE-FFQ1 and IODINE-FFQ2 intakes was significant (*p* = 0.0000; R = 0.8105).

**Figure 2:**
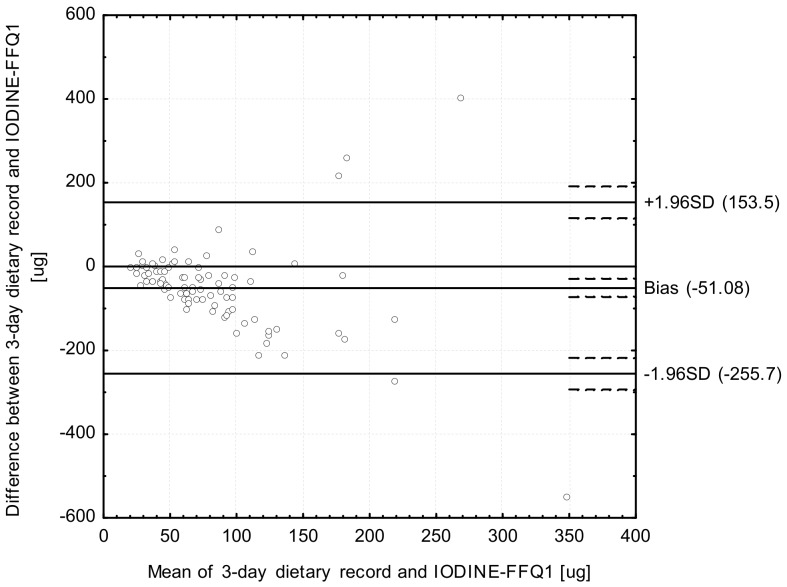
Bland-Altman plot comparing IODINE-FFQ1 with 3-day dietary record iodine daily intake (Bland-Altman index of 5.5%). IODINE-FFQ1—food frequency questionnaire filled out directly after conducting 3-day dietary record.

The Bland-Altman plot comparing IODINE-FFQ1 with the 3-day dietary record daily iodine intake is presented in Figure 2. The mean absolute difference in iodine intake was observed to amount to −51.08. After adding ±1.96 standard deviation for the LOA, an interval from −255.7 (lower agreement limit) to 153.5 (upper agreement limit) was obtained. The number of individuals observed to be beyond the LOA value was 85 out of 90, which confirmed a Bland-Altman index of 5.5%.

**Figure 3:**
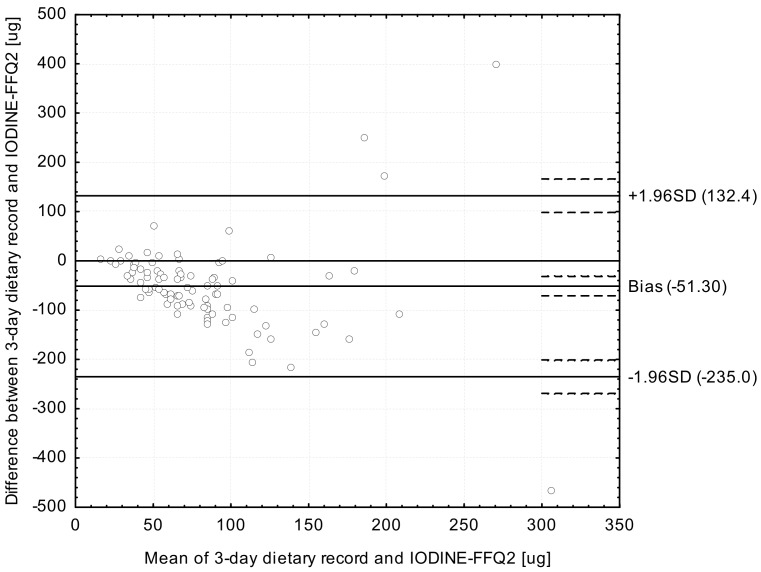
Bland-Altman plot comparing IODINE-FFQ2 with 3-day dietary record iodine daily intake (Bland-Altman index of 4.4%). IODINE-FFQ2—food frequency questionnaire filled out 6 weeks after IODINE-FFQ1.

The Bland-Altman plot comparing IODINE-FFQ2 with the 3-day dietary record daily iodine intake is presented in Figure 3. The mean absolute difference in iodine intake was observed to amount to −51.30. After adding ±1.96 standard deviation for the LOA, an interval from −235.0 (lower agreement limit) to 132.4 (upper agreement limit) was obtained. The number of individuals observed to be beyond the LOA value was 86 out of 90, which confirmed a Bland-Altman index of 4.4%.

**Figure 4:**
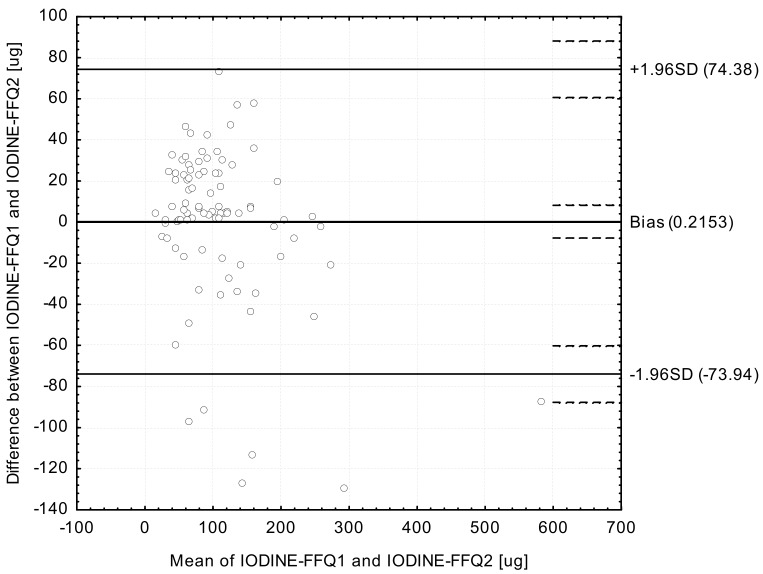
Bland-Altman plot comparing IODINE-FFQ1 with IODINE-FFQ1 iodine daily intake (Bland-Altman index of 6.7%). IODINE-FFQ1—food frequency questionnaire filled out directly after conducting 3-day dietary record; IODINE-FFQ2—food frequency questionnaire filled out 6 weeks after IODINE-FFQ1.

The Bland-Altman plot comparing IODINE-FFQ1 with IODINE-FFQ2 daily iodine intake is presented in Figure 4. The mean absolute difference in iodine intake was observed to amount to 0.2153. After adding ±1.96 standard deviation for the LOA, an interval from −73.94 (lower agreement limit) to 74.38 (upper agreement limit) was obtained. The number of individuals observed to be beyond the LOA value was 84 out of 90, which confirmed a Bland-Altman index of 6.7%.

## 4. Discussion

### 4.1. Iodine Intake 

It is indicated that women characterized by a fish intake of less than two servings per week and a dairy product intake of less than 2 servings per day have significantly lower iodine intakes than those who consume the recommended intake of fish and dairy products [33]. In the case of the countries of Northern Europe–Norway [34] and Iceland [35], fish intake contributes to the high share of iodine intake. On the other hand, in Poland, the fish intake is low or even very low, contributing not only to low iodine intake, being observed in the presented study and other studies [36], but also to low intake of n-3 polyunsaturated fatty acids [37] and low intake of vitamin D [22] in women. Taking it into account, due to the higher intake, rather dairy products may contribute to higher iodine intake, similarly as it is indicated for Denmark [38]. However, also in Norway, it was indicated, that association between fish and seafood intake and iodine nutritional status is rather weak, that is attributed *inter alia* to applied food fortification or supplementation [39,40].

According to the WHO [1] analysis, the recommended strategy for global iodine deficiency disorders, should include correcting deficiency by increasing iodine intake through supplementation or food fortification. Similarly, the World Bank [41] recommended fortification with iodine, indicating, that no other technology offers as large an opportunity to improve lives at such low cost and in such a short time, as micronutrient programmes [9]. The iodine fortification is the most commonly applied in the case of salt [1], but in some countries also bread [42], milk [43] and water [44] are iodine-fortified.

In a number of studies it was stated, that iodine nutritional status may be improved after implementing obligatory fortification, e.g., in Slovenia adequate iodine intake is attributed mainly to excessive intake of fortified salt [45]. However, even in such situation, when obligatory fortification is applied, the iodine intake often does not obtain an expected level, but still a moderate one [46,47] and some groups are characterized by inadequate iodine intake [38]. Taking it into account, it is indicated, that constant iodine intake monitoring should be a priority to ensure the sufficient intake [48]. The urgent need for a properly planned public health strategies is especially indicated in the case of women of reproductive age, in particular pregnant ones [49], being highly vulnerable to iodine deficiency and sensitive for thyroid function disturbances [50].

### 4.2. Comprehensive Food Frequency Questionnaires to Assess Iodine Intake

It is commonly recognized that food frequency questionnaires provide a useful way to assess the dietary intake for groups, due to low burden on respondents and investigators [51], however, for a number of population groups there is a lack of iodine questionnaires of proven validity [52].

The food frequency questionnaires may be easy method to use, to obtain the estimation of nutrients intake, as the multiple-day dietary records should be applied only for highly-motivated individuals, due to general low motivation to conduct [53] and results of 3-day dietary records may be also unreliable, due to within-person variation of day-to-day dietary intake [54]. However, to obtain a practical tool to use in a large population, questionnaires should be as short and simple as possible so as to be associated with an actual low burden. 

According to the report of a joint Food and Agriculture Organization of the United Nations (FAO) and WHO consultation [55], the food frequency questionnaires are divided into two groups —brief food frequency questionnaires to assess the intake of one or several specific nutrients and comprehensive food frequency questionnaires designed to estimate an intake of a large number of nutrients (multiple-nutrients questionnaire). The type of questionnaire determines the number of questions associated with specific food products–comprehensive food frequency questionnaires generally include 50–150 food products, while the brief food frequency questionnaires are characterized by reduced food products list, so the choice of food products listed and the number of them may be a crucial issue [55]. It must be emphasized, that to avoid other limitations, the food frequency questionnaires must be not only short, but also easy to understand for respondents, and must avoid any complexity of the food products list [56]. 

A number of a comprehensive food frequency questionnaires, assessing *inter alia* the iodine intake, is used in various studies [12,57,58]. In the study of Moreira et al. [59] and Sauvageot et al. [60], the significant correlations were stated for iodine intake, assessed on the basis of applied food frequency questionnaires, including respectively 82 and 135 various food items, and respectively iodine intake assessed on the basis of the 4-day dietary record [59] and iodine status [60]. The iodine status in the study of Sauvageot et al. [60] was assessed on the basis of iodine/creatinine ratio, indicating, that there is a possibility to assess accurately iodine status, on the basis of food frequency questionnaire and the existing association between iodine intake and status was also confirmed in the study of Kim et al. [61], for the urinary iodine excretion.

In the study of Feng et al. [62], it was stated, that for an Internet-based Diet Questionnaire for Chinese (IDQC), including 135 various food items, in comparison with the 3-day dietary record, the share of individuals classified into the same or adjacent quartile was for iodine 100%. In the study of Ogawa et al. [63], in similar comparison with the 3-day dietary record, for the food frequency questionnaire, including 167 various food items, the share of individuals classified into the same or adjacent quintile was for iodine 61.2%. In the presented own study, it was 86.67% and 83.33%, for respectively IODINE-FFQ1 and IODINE-FFQ2, while classified into tertiles, but the questionnaire included only 44 various food items.

However, the study of Mouratidou et al. [64] revealed, that validated multiple-nutrients Sheffield Food Frequency Questionnaire underestimated iodine intake, in comparison with 24-h dietary recall, that is untypical, as food frequency questionnaires are rather prone to overestimation [48]. Also, in the study of Khalesi et al. [65] it was stated, that for validated multiple-nutrients food frequency questionnaire, Bland-Altman plots and linear regression results revealed, that it may not be a valid tool estimating potassium, iron and iodine intakes. In the case of such nutrients, the specific brief questionnaires may be of a great value in order to guarantee the proper estimation of intake.

In the study of Zhang et al. [66], the reason of low validity, of applied multiple-nutrients food frequency questionnaire, in the case of iodine, was discussed. It was stated, that as in China the iodine-fortified salt is one of the main sources of mentioned nutrient [67], the applied questionnaire, must have included question about the frequency and amount of consumed fortified salt, instead of just a command to “select (…) taste as light, medium or strong” [66]. Always, if the iodine intake is planned to be assessed, both in the case of the comprehensive food frequency questionnaires and of the brief food frequency questionnaires, it is essential to include the fortified products. In the case of Polish diet it was the salt with 114.6 µg of iodine per 5 g teaspoon, that was included into designed IODINE-FFQ, while in the study of Štimec et al. [68], during adjusting the Harvard University Food Frequency Questionnaire developed by Willett [69], the 10 types of table salt available on the Slovenian market were included.

### 4.3. Validation of Brief Iodine-Specific Food Frequency Questionnaire

In order to obtain a practical tool dedicated exclusively for iodine which can assess its intake in the quickest possible way, fewer questions must be planned, than in the multiple-nutrients comprehensive questionnaires, and as a result, a brief questionnaire may be associated with lower overestimation [70]. While designing the brief food frequency questionnaire, the number of food products should be reduced carefully, bearing in mind that the association between the applied food products list and the obtained results is stated to be one of the major sources of errors in the food frequency questionnaires [71]. In some cases, the food products list is very limited, so number of questions is also reduced—such approach was planned in the study of Dahl et al. [72] as in the iodine-specific food frequency questionnaire only fish and dairy products were included, and it was established that from other sources the typical iodine intake is 30 µg per day. Similarly, in the study of Rasmussen et al. [8], in the assessment based on iodine-specific food frequency questionnaire, it was stated, that dairy products, other beverages and fish intake contribute 86% of total iodine intake in Danish population, while 53 food items high in iodine were included into questionnaire. However, all food frequency questionnaires must be designed for specific populations that may be observed during analysis of iodine-specific food frequency questionnaire for Japanese population, in which 14 types of algae products were included, as well as 14 types of fish and shellfish [73]. Taking it into account, it must be indicated, that it is important to obtain the specific questionnaires designed for such populations, for which questionnaires were not created so far. 

Each obtained food frequency questionnaire must be validated, but while analyzing the results of validations obtained by the authors of questionnaires, it seems that various methods are applied and results often vary. In the study of Taib and Isa [17], for analysis of the iodine intake assessed on the basis of semi-quantitative food frequency questionnaire (not described in detail) and 24-h dietary recall, the correlation was very strong, indicating almost perfect correlation (R = 0.954). However, such level is higher than commonly observed level of 0.3–0.6 [12] and higher than defined by Willett “ceiling of validity” of R = 0.7 (level above which R values are very rare in the validation of food frequency questionnaires) [74], so such a high level may be attributed to specific group analysed or specific methodology applied, and lower levels should also be perceived as a positive validation. In the study of Brantsæter et al. [75,76], the correlation between iodine intake obtained from multiple-nutrients Norwegian Mother and Child Cohort Study food frequency questionnaire (MoBa FFQ) and 4-day weighted dietary record was lower (R = 0.46), similarly as in the presented own study between IODINE-FFQ and 3-day dietary record in which significantly lower values were observed. 

In the case of such food frequency questionnaires, that enable quick assessment of intake to observe changes caused by nutritional counseling, the high repeatability of questionnaire is a priority. It must be indicated, that the reproducibility obtained for elaborated IODINE-FFQ (R = 0.8105) was even higher than in the multiple-nutrients comprehensive questionnaire, for pregnant women, in the study of Vioque et al. [77], even though for iodine, R = 0.70 was the highest of obtained for all assessed nutrients.

The Bland-Altman plot is indicated as the “gold standard” in the validation [15], and it is emphasized, that to assess both validity and reproducibility, this method should be used instead of correlation, while other methods may be also used, but only in conjunction with the Bland-Altman method and only kappa statistics is indicated as a method that may be used instead, but only for measures involving small numbers of ordered categories [78]. Taking it into account, the results of Bland-Altman plot must be mainly described and treated as primary results, while other should be treated as just supporting ones.

However, while analyzing the results of Bland-Altman plot for validated iodine-specific food frequency questionnaires, it must be indicated, that values of Bland-Altman index higher than 5% are commonly obtained and also accepted, even if in general values lower than 5% are expected [32]. In the study of Rasmussen et al. [13], the analysis of Bland-Altman plot revealed larger difference between results of iodine-specific food frequency questionnaire and dietary records, with increased iodine intake, but without any systematic deviations between compared methods. In the study of Combet and Lean [15], the number of individuals observed to be beyond the LOA value was 41 out of 43 (Bland-Altman index of 4.6%), but in the study of Condo et al. [16] it was 91 out of 96 (Bland-Altman index of 5.2%) for the comparison of the results of iodine-specific food frequency questionnaires and dietary records. The indicated results are comparable with the results of own study, as in the similar comparison, the Bland-Altman index of 5.5% and 4.4% were observed for IODINE-FFQ1 and IODINE-FFQ2, respectively. Consequently, it must be indicated, that the results obtained for IODINE-FFQ must be perceived as satisfactory. 

Taking into account especially high reproducibility, confirmed for all applied methods of statistical analysis, the main aim of the validated IODINE-FFQ should be application in the case of repeated assessments in the period of time, e.g., after conducting dietary education. Moreover, the obtained questionnaire, due to its simplicity may be used in a larger groups of individuals, which allows analyzing the intake in populations and indicating individuals characterized by especially low intake. However, the obtained questionnaire, as a dietary assessment method, may be used also in other situations, to obtain assessment of iodine intake, conducted by dietitians or other public health specialists.

While analyzing the results of the conducted study, the discrepancy between the results of the Bland-Altman method, indicated as a “gold standard” and kappa statistics must be explained. It results from the fact, that Bland-Altman method analyses absolute iodine intake, while kappa statistics—tertiles of iodine intake. As a result, in the conducted study, the Bland-Altman plot was based on the primal data, and kappa statistics, on recalculated ones, that may had resulted in changing of the observed associations. However, in order to present the full picture, all the results were presented, but the Bland-Altman plot was emphasized to be the dominant result.

In spite of the fact, that the results of the Bland-Altman plot may be interpreted as a positive validation of the analysed IODINE-FFQ, the limitations of the study must be indicated. The main limitation is associated with the fact, that IODINE-FFQ, being self-reported method was validated using 3-day dietary record, that is also self-reported one. It may contribute to systematic error, that must be considered in the case of all the validation studies assessing the nutrients dietary intake questionnaires. However, as was previously indicated, validation conducted using the data of nutrient status, instead of intake, contribute to the possibility of misinterpreting the data and must be very carefully planned and conducted. As a result, the further studies should include also the analysis of iodine status, to indicate if the assessed questionnaire could allow to conclude also about iodine status, or the iodine intake only. Moreover, the obtained questionnaire must be still analysed and validated in order to assess the possibility of using it also in other population groups or in other countries. While questionnaires are used in other countries, than the designing was conducted, the adjustment is needed, that includes incorporating additional questions about iodine-fortified products. Taking it into account, the obtained IODINE-FFQ may be applied also for other countries of Eastern Europe, characterized by a similar diet, as Poland, but only after a necessary adjustments including iodine-fortified products. 

## 5. Conclusions

Assessment of IODINE-FFQ revealed a satisfactory level of validity and reproducibility in the analysis of Bland-Alman plot. The IODINE-FFQ may be indicated as a tool for the assessment of iodine intake in the young women in Poland, however further studies should be considered in order to obtain the practical tool for public health specialists. Due to the lack of validated iodine-specific food frequency questionnaires for countries of Eastern Europe, the IODINE-FFQ may be adjusted for courtiers other than Poland including iodine-fortified products.

## Figures and Tables

**Table 1:** The scheme of an applied food frequency questionnaire including food products, serving sizes and frequencies in the IOdine Dietary INtake Evaluation-Food Frequency Questionnaire—IODINE-FFQ.

Group of Products	Products	Serving Size	Number of Servings per Week
Dairy products	Whet, sheep milk	300 g (large glass)	
	Milk and milk beverages (yoghurt, kefir, buttermilk, cream)	300 g (large glass)	
	Milk powder	10 g (tablespoon)	
	Condensed milk	10 g (tablespoon)	
	Camembert, brie cheese	150 g (packaging)	
	Rennet cheese	20 g (thin slice)	
	Cottage cheese	200 g (packaging)	
	Curd cheese, cream cheese spread	30 g (thin slice, tablespoon)	
	Fromage frais	150 g (packaging)	
	Processed cheese	25 g (slice, triangle serving)	
Eggs		50 g (egg)	
Meat	Offal, lamb	100 g (palm of small hand)	
	Veal		
	Other types of meat		
	Cold cuts	15 g (thin slice of ham, 1/3 of wiener)	
Fish	Cod, pollock	100 g (palm of small hand)	
	Plaice, halibut, tuna, mackerel, salmon		
	Flounder, herring, sole, sardine		
	Trout, pike, perch, eel, carp		
	Smoked eel	50 g (half of palm of small hand)	
	Other smoked fishes		
	Fish products in tins and pickled herring	50 g (half of small tin, rollmop)	
	Herring in a creamy sauce	50 g (2 tablespoons)	
Fats		10 g (tablespoon)	
Cereal products	Crispbread	10 g (slice)	
	Other types of bread	30 g (slice, half of a roll)	
	Wheat bran	5 g (tablespoon)	
	Cereals, cereal grains	5 g (tablespoon)	
	Rice, pasta, groats	100 g of cooked (glass)	
Vegetables	Broccoli, spinach	100 g (half of a glass, 1 glass of leafy vegetables)	
	Radish, turnip, asparagus, broad bean, kale, green peas, chives		
	Other vegetables		
Legumes	Peas	15 g of dry (tablespoon)	
	Other dry legumes		
Potatoes		50 g (2 tablespoons of puree)	
Fruits		100 g (half of a glass)	
Nuts and seeds	Hazelnuts, peanuts	30 g (handful)	
	Other		
Beverages	Coffee, tea	250 g (glass)	
	Fruit juices, beer		
	Wine	150 g (wineglass)	
Other	Chocolate	20 g (3–4 chocolate bar squares)	
	Iodine-fortified salt	5 g (teaspoon)	
	Gelatin		

**Table 2:** The content of iodine in one serving of a size specified in the IODINE-FFQ.

Group of Products	Products	Serving Size	Iodine Content/Serving (µg)
Dairy products	Whet, sheep milk	300 g (large glass)	27.0
	Milk and milk beverages (yoghurt, kefir, buttermilk, cream)	300 g (large glass)	10.2
	Milk powder	10 g (tablespoon)	3.2
	Condensed milk	10 g (tablespoon)	1.2
	Camembert, brie cheese	150 g (packaging)	17.4
	Rennet cheese	20 g (thin slice)	6.9
	Cottage cheese	200 g (packaging)	20.0
	Curd cheese, cream cheese spread	30 g (thin slice, tablespoon)	1.0
	Fromage frais	150 g (packaging)	4.2
	Processed cheese	25 g (slice, triangle serving)	1.8
Eggs		50 g (egg)	4.7
Meat	Offal, lamb	100 g (palm of small hand)	3.3
	Veal		2.2
	Other types of meat		1.3
	Cold cuts	15 g (thin slice of ham, 1/3 of wiener)	2.1
Fish	Cod, pollock	100 g (palm of small hand)	94.4
	Plaice, halibut, tuna, mackerel, salmon		48.6
	Flounder, herring, sole, sardine		26.3
	Trout, pike, perch, eel, carp		5.6
	Smoked eel	50 g (half of palm of small hand)	2.2
	Other smoked fishes		33.3
	Fish products in tins and pickled herring	50 g (half of small tin, rollmop)	23.9
	Herring in a creamy sauce	50 g (2 tablespoons)	3.9
Fats		10 g (tablespoon)	0.3
Cereal products	Crispbread	10 g (slice)	1.4
	Other types of bread	30 g (slice, half of a roll)	0.7
	Wheat bran	5 g (tablespoon)	1.6
	Cereals, cereal grains	5 g (tablespoon)	0.2
	Rice, pasta, groats	100 g of cooked (glass)	0.7
Vegetables	Broccoli, spinach	100 g (half of a glass, 1 glass of leafy vegetables)	13.5
	Radish, turnip, asparagus, broad bean, kale, green peas, chives		5.9
	Other vegetables		2.1
Legumes	Peas	15 g of dry (tablespoon)	2.1
	Other dry legumes		0.6
Potatoes		50 g (2 tablespoons of puree)	1.5
Fruits		100 g (half of a glass)	1.6
Nuts and seeds	Hazelnuts, peanuts	30 g (handful)	15.0
	Other		4.1
Beverages	Coffee, tea	250 g (glass)	2.5
	Fruit juices, beer		1.5
	Wine	150 g (wineglass)	52.5
Other	Chocolate	20 g (3–4 chocolate bar squares)	0.8
	Iodine-fortified salt	5 g (teaspoon)	114.6
	Gelatin		0.2

**Table 3:** The iodine intake calculated using 3-day dietary record and IODINE-FFQ, accompanied by share of individuals characterized by adequate or inadequate intake.

			3-Day Dietary Record	IODINE-FFQ1	IODINE-FFQ2
Mean (µg)			57.90	108.98	109.19
Standard deviation (µg)			65.78	86.28	72.95
Median (µg)			40.02 *	90.06 *	97.34 *
Minimum (µg)			4.06	12.37	15.36
Maximum (µg)			469.18	625.11	537.95
Share of individuals characterized in comparison with recommendation by Jarosz [25]	adequate intake	*n*	9	41	46
		(%)	10.00	45.46	51.11
	inadequate intake	*n*	81	49	44
		(%)	90.00	54.44	48.89

* Distribution different than normal (verified using Shapiro-Wilk test—*p* ≤ 0.05); IODINE-FFQ1—food frequency questionnaire filled out directly after conducting 3-day dietary record; IODINE-FFQ2—food frequency questionnaire filled out 6 weeks after IODINE-FFQ1.

**Table 4:** The number and share of individuals classified into the same tertile and misclassified, as well as individuals of the same or conflicting iodine intake adequacy category in comparison of 3-day dietary record and IODINE-FFQ.

			IODINE-FFQ1 vs. 3-Day Dietary Record	IODINE-FFQ2 vs. 3-Day Dietary Record	IODINE-FFQ1 vs. IODINE-FFQ2
Individuals classified into the same tertile		*n*	34	31	65
		%	37.78	34.44	72.22
Individuals misclassified (classified into opposite tertiles)		*n*	12	15	1
		%	13.33	16.67	1.11
Weighted *κ* statistic			0.15	0.075	0.675
Individuals of the	same iodine intake adequacy category	*n*	56	47	77
		%	54.90	52.22	85.56
	conflicting iodine intake adequacy category	*n*	46	43	13
		%	45.10	47.78	14.44

IODINE-FFQ1—food frequency questionnaire filled out directly after conducting 3-day dietary record; IODINE-FFQ2—food frequency questionnaire filled out 6 weeks after IODINE-FFQ1.
